# Presumed acute posterior multifocal placoid pigmentary epitheliopathy associated with *Bartonella* infection

**DOI:** 10.5935/0004-2749.2022-0032

**Published:** 2023-03-20

**Authors:** Maianna Macedo de Sousa, Claudio Zett, João Carlos Diniz Arraes, Luiz H. Lima

**Affiliations:** 1 Universidade Federal do Tocantins, Araguaína, TO, Brazil; 2 Pontificia Universidad Católica de Valparaíso, Valparaíso, Chile; 3 Universidade Federal de São Paulo, São Paulo, SP, Brazil

**Keywords:** Acute posterior multifocal placoid pigment epitheliopathy, *Bartonella quintana*, *Bartonella henselae*, Cat-scratch disease, Epiteliopatia pigmentar placoide multifocal posterior aguda, *Bartonella quintana*, *Bartonella henselae*, Doença da arranhadura de gato

## Abstract

To report a unique case of acute posterior multifocal placoid pigment epitheliopathy (APMPPE) in a patient with positive serology for *Bartonella*, presenting with ocular signs and symptoms not attributable to other diseases. A 27-year-old woman presented with decreased visual acuity in both eyes. Multimodal fundus image analysis was performed. A color fundus photograph of both eyes revealed peripapillary and macular yellow-white placoid lesions. The fundus autofluorescence of both eyes demonstrated hypo- and hyperautofluorescence of the macular lesions. Fluorescein angiography showed early-stage hypofluorescence and late staining of placoid lesions in both eyes. Spectral domain optical coherence tomography (SD-OCT) of both eyes revealed irregular elevations in the retinal pigment epithelium with the disruption of the ellipsoid zone on the topography of macular lesions. At 3 months after the treatment initiation for *Bartonella* infection, the placoid lesions became atrophic and hyperpigmented, and SD-OCT revealed loss of both the outer retinal layers and retinal pigment epithelium on the topography of macular lesions in both eyes.

## Abstract (pt)

Caso de epiteliopatia pigmentada placoide multifocal posterior aguda presumida em paciente com sorologia positiva para *Bartonella*. Paciente feminina de 27 anos apresentou diminuição da acuidade visual em ambos os olhos. Análise multimodal de imagem foi realizada. A retinografia mostrou revelou lesões placoides amarelo-esbranquiçadas nas áreas peripapilar e macular de ambos os olhos. A autofluorescência demonstrou hipo e hiperautofluorescência em ambos os olhos, na mesma topografia das lesões detectadas na retinografia. A angiofluoresceínografia mostrou hipofluorescência na fase inicial do exame e hiperfluorescência tardia das lesões placoides em ambos os olhos. A tomografia de coerência óptica de domínio espectral de ambos os olhos revelou elevações irregulares do epitélio pigmentado da retina com descontinuação da zona elipsoide na área macular. Três meses após o início do tratamento para infecção por Bartonella, as lesões placoides tornaram-se atróficas e hiperpigmentadas, e a tomografia de coerência óptica revelou perda das camadas externas da retina e do epitélio pigmentado da retina na topografia das lesões maculares em ambos os olhos.

## INTRODUCTION

Acute posterior multifocal placoid pigment epitheliopathy (APMPPE) is an idiopathic chorioretinopathy classified as a white-dot syndrome that was first described by Gass in 1968^(1)^. It is often bilateral and self-limited (visual symptoms resolve by 4-8 weeks), and affects middle-aged men and women equally. Nearly one-third of patients reported previous flu-like symptoms before the onset of APMPPE. They generally notice a decresead vision in association with central and paracentral scotomas^(2)^.

APMPPE represents an inflammation of the retinal pigment epithelium (RPE) and outer retina that appears as placoid lesions during the acute phase and RPE hyperpigmentation and atrophy in later stages^(2)^. Although no specific laboratory examination is recommended for diagnosis, fluorescein (early hypofluorescence corresponding to the placoid lesions, followed by late hyperfluorecent staining) and indocyanine green angiography (early and late hypofluorescence corresponding to placoid lesions) imaging characteristics are typical of this disease. Optical coherence tomography (OCT) and fundus autofluorescence (FAF) may also contribute to the diagnosis of this condition. Hypoautoflourescence and hyperreflectivity of the outer layers corresponding to the placoid lesions are observed on FAF and OCT, respectively^(2)^.

APMPPE can have associated systemic diseases, such as sarcoidosis, ulcerative colitis, erythema nodosum, cerebral vasculitis and granulomatosis with polyangiitis. Viral and bacterial infectious diseases, such as adenovirus, hepatitis B, Lyme disease, mumps, group A streptococcus, coxsackievirus B, and tuberculosis, have also been reported to be related to APMPPE^(3)^. The purpose of this case report is to describe a unique case of APMPPE-like lesions in a patient who tested positive for *Bartonella*.

## CASE REPORT

A 27-year-old woman presented to the clinic complaining of blurred vision in both eyes for 2 weeks. She did report recent flu-like symptoms, such as fever, malaise, and headaches. Recent contact with animals, including cats, was not reported. The patient had an unremarkable previous medical and ocular history.

**Figure 1:**
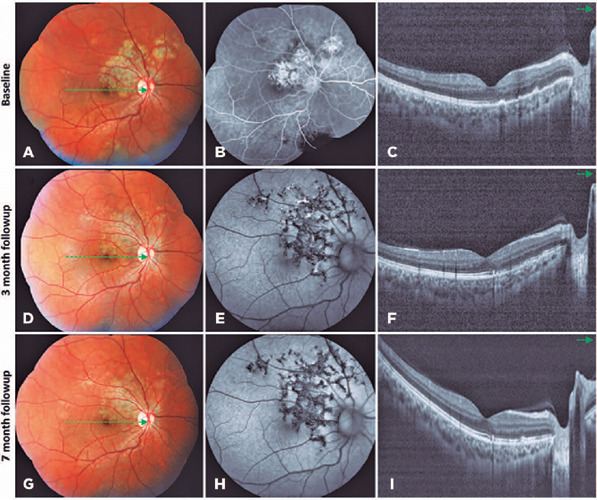
Multimodal imaging of the right eye. A, B, and C. At baseline, color fundus photograph (A) revealed whitish-yellow placoid lesions in the peripapillary and macular areas. Fluorescein angiography (B) showed late staining of the placoid lesions. Spectral-domain optical coherence tomography (SD-OCT) revealed irregular elevations associated with loss at the retinal pigment epithelium (RPE) level and overlying disruption of the ellipsoid zone (EZ) on the topography of the placoid lesions (C). D, E, and F. At 3 months after treatment initiation, the placoid lesions presented with some degree of atrophy and hyperpigmentation (D). Fundus autofluorescence (FAF) demonstrated hypoautofluorescence of the healed lesions (E), and SD-OCT revealed partial reorganization of the outer retinal layers and RPE on the topography of macular lesions (F). G, H, and I. At the 7-month follow-up, the peripapillary and macular placoid lesions became more atrophic and hyperpigmented (G) and presented with hypoautofluorescence on FAF (H). SD-OCT revealed complete reorganization of the outer retinal layers and RPE within the macular area (I).

**Figure 2:**
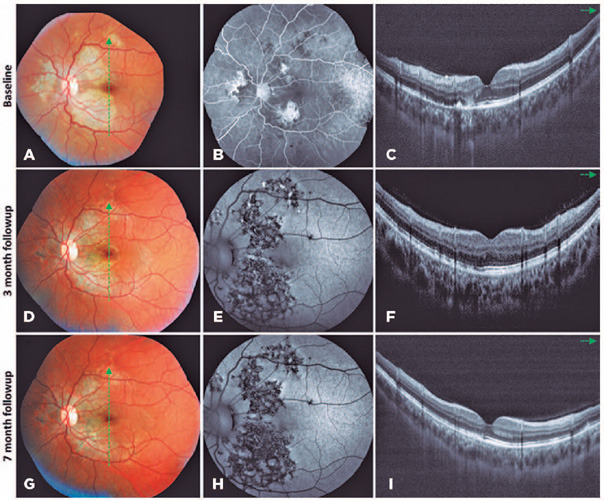
Multimodal imaging of the left eye. A, B, and C. At baseline, color fundus photograph (A) revealed whitish-yellow placoid lesios in the peripapillary and macular areas. Fluorescein angiography (B) showed late staining of the placoid lesions. Spectral-domain optical coherence tomography (SD-OCT) revealed irregular elevations associated with loss at the retinal pigment epithelium (RPE) level and overlying disruption of the ellipsoid zone (EZ) on the topography of placoid lesions (C). D, E, and F. At 3 months after treatment initiation, the peripapillary and macular placoid lesions presented with some degree of atrophy and hyperpigmentation (D). FAF demonstrated hypoautofluorescence of the healed lesions (E), and SD-OCT revealed partial reorganization of the outer retinal layers and RPE on the topography of macular lesions (F). G, H, and I. At the 7-month follow-up, the peripapillary and macular placoid lesions became more atrophic and hyperpigmented (G) and presented with hypoautofluorescence on fundus autofluorescence (H). SD-OCT revealed complete reorganization of the outer retinal layers and RPE within the macular area (I).

On ocular examination, the best-corrected visual acuity (BCVA) was 20/30 in both eyes. Pupillary reactions, slit-lamp biomicroscopy of the anterior segment, and intraocular pressure were normal in both eyes. The color fundus photograph (TRX 50 DX, Topcon Medical Systems, Tokyo, Japan) of both eyes revealed deep whitish-yellow placoid lesions in peripapillary and macular areas. Optic disc edema and macular exudation were not observed in both eyes. Fluorescein angiography (TRX 50 DX, Topcon Medical Systems) showed early hypofluorescence along with late staining of the placoid lesions in both eyes. fundus autofluorescence (TRX 50 DX, Topcon Medical Systems) demonstrated hypoautofluorescence of the healed lesions and hyperautoflurescence of the active lesions. Spectral-domain OCT (RTVue-XR Avanti, Optovue Inc, Fremont, CA, USA) revealed irregular elevations at the RPE level with overlying the disruption of the ellipsoid zone (EZ) on the topography of macular lesions in both eyes (Figures 1 and 2).

Laboratory tests revealed normal blood counts CBC. Chest and sinus X-ray image findings were also normal. Serologies for cytomegalovirus, herpes simplex virus, human immunodeficiency virus, *Histoplasma capsulatum, Toxoplasma gondii, Toxocara canis,* and *Borrelia burgdorferi* were negative. *Bartonella quintana* indirect fluorescent antibody (IFA) titers were positive for IgM at 1:200 (reference normal range, <1:20) and IgG at 1:1280 (reference normal range, IgG: <1:128). A *B. henselae* IgG antibody reaction (1:640) (reference normal range, IgG: <1:128) associated with a non-reactive IgM antibody was also observed.

Doxycycline at 100 mg twice daily and prednisone (0.5mg/kg/day) were initiated. Three months after the treatment initiation, the BCVA improved to 20/20 in both eyes, the peripapillary and macular placoid lesions became atrophic and hyperpigmented, and the spectral domain optical coherence tomography (SD-OCT) revealed loss of both the outer retinal layers and RPE on the topography of macular lesions in both eyes (Figures 1 and 2).

## DISCUSSION

APMPPE represents an uncommon disease characterized by the sudden presentation of yellow-white inflammatory lesions at the RPE level and choriocapillaris. The characteristic APMPPE fundus finding of placoid whitish- yellow deep retinal lesions and usual pattern of early hypofluorescence and late hyperfluorescence corresponding to the placoid lesions on FA were detected in the present case. The typical APMPPE OCT findings of irregularities in the EZ and RPE and the hypo- and hyperautofluorescence of chronic and active lesions were also observed. APMPPE should be differentiated from other conditions that cause deep chorioretinitis, including acute syphilitic posterior placoid chorioretinopathy, serpiginous choroiditis, and acute occult zonal external retinopathy^(4)^. The negative serology for syphilis, multimodal imaging characteristics of placoid lesions, and natural disease course are clinical data that make less probable these three disorders as the diagnosis for the present case.

Bartonellosis or cat-scratch disease may appear in a broad range of systemic presentations, including as hepa titis, endocarditis, meningitis, encephalopathy, and hemolytic anemia^(5)^. Ocular bartonellosis is rare, and neuroretinitis corresponds to the majority of cases with fundus affection. *Bartonella henselae* is the most common *Bartonella* species infecting humans, and there are only very cases of neuroretinitis associated with *Bartonella quintana* were reported^(6)^. In the present case, *Bartonella* infection was confirmed by positive serology for *Bartonella quintana* and *Bartonella henselae*. A diagnosis of *Bartonella quintana* was made after elevated IgM and IgG titers 1:200 and 1:1280, respectively were found on the IFA test. *B. henselae* IgG antibody reaction was positive at 1:640. A positive IFA IgM (titer >1:20) suggests a current infection with either *Bartonella henselae* or *Bartonella quintana*. IgG titers >1:256 are considered strongly suggestive of recent infection. Normal serum specimens usually have an IgG titer of <1:128^(7)^.

*B. henselae* and *B. quintana* are closely related *Bartonella* species that induce cross-reactivity when human sera is tested using an IFA assay^(8)^. The IgM antibodies to *Bartonella* species are commonly observed early in infection with decreased titers 8-10 weeks thereafter, advocating that IgM antibodies may not be suitable during the later stages of the cat-scratch disease^(9)^. Remarkably, a separate positive IgM antibody result should be considered carefully and interpreted in conjunction with the timing of the potential exposure and duration of patient symptoms. A new IFA testing performed 3 weeks later is recommended to show the seroconversion of anti-*Bartonella* IgG^(9)^. Within 3 months of treatment with doxycycline, the patient’s visual acuity improved, and retinal lesion activity decreased. Although our patient did not have contact with cats, other vectors, such as birds, ticks, and flying insects, may carry flea feces, so that *Bartonella* inoculation in humans may occur through a small open wound or even in the mucous membranes^(10)^.

The pathogenesis of APMPPE is still unclear, and a hypothesis is that there is a vascular involvement damaging the choroid that can lead to a partial choroidal ischemia, results in RPE abnormalities, and subsequently alters the photoreceptors. Also, it is feasible that an initial mechanism affecting the outer retina and RPE may secondarily induce choroidal changes. Furthermore, systemic associations of APMPPE suggest an intrinsic vasculitis^(11)^. Although the exact APMPPE etiology is unknown, flu-like symptoms are observed in up to 50% of APMPPE cases, and associations with viral diseases such as mumps, adenovirus, and coxsackievirus B were reported. APMPPE related to bacterial infections, including Lyme disease and group A streptococcus, has also been described^(2,3)^. A delayed-type hypersensitivity reaction may justify all these associations. These infectious agents may trigger an immune reaction and stimulate sensitized T lymphocytes. Consequently, macrophages and cytotoxic T cells are activated by the released lymphokines^(11)^. *B. quintana* and *B. henselae* are gram-negative bacilli that have the potential to act as an infectious trigger provoking an immune reaction. Both *Bartonella* species can cause invasion in endotelial cells, resulting in inflammatory response, vasoproliferation, and obstructive vasculitis^(12)^. Therefore, this vascular insult introduced by *Bartonella* may be related to the APMPPE lesions appearance in the present case since it could affect the choroid and cause partial choroidal ischemia, leading to the hypoperfusion of the terminal choroidal lobules in the posterior pole with secondary injury of the RPE and retinal outer layers. To the best of our knowledge, this is the first report of APMPPE associated with *Bartonella* infection.
